# Fusion Learning for sEMG Recognition of Multiple Upper-Limb Rehabilitation Movements

**DOI:** 10.3390/s21165385

**Published:** 2021-08-09

**Authors:** Tianyang Zhong, Donglin Li, Jianhui Wang, Jiacan Xu, Zida An, Yue Zhu

**Affiliations:** College of Information Science and Engineering, Northeastern University, Shenyang 110819, China; 1970815@stu.neu.edu.cn (T.Z.); 1910309@stu.neu.edu.cn (D.L.); 1410330@stu.neu.edu.cn (J.X.); 1900753@stu.neu.edu.cn (Z.A.); 20174129@stu.neu.edu.cn (Y.Z.)

**Keywords:** surface electromyogram, motion intention recognition, multiscale time–frequency information fusion representation, multiple feature fusion network, deep belief network

## Abstract

Surface electromyogram (sEMG) signals have been used in human motion intention recognition, which has significant application prospects in the fields of rehabilitation medicine and cognitive science. However, some valuable dynamic information on upper-limb motions is lost in the process of feature extraction for sEMG signals, and there exists the fact that only a small variety of rehabilitation movements can be distinguished, and the classification accuracy is easily affected. To solve these dilemmas, first, a multiscale time–frequency information fusion representation method (MTFIFR) is proposed to obtain the time–frequency features of multichannel sEMG signals. Then, this paper designs the multiple feature fusion network (MFFN), which aims at strengthening the ability of feature extraction. Finally, a deep belief network (DBN) was introduced as the classification model of the MFFN to boost the generalization performance for more types of upper-limb movements. In the experiments, 12 kinds of upper-limb rehabilitation actions were recognized utilizing four sEMG sensors. The maximum identification accuracy was 86.10% and the average classification accuracy of the proposed MFFN was 73.49%, indicating that the time–frequency representation approach combined with the MFFN is superior to the traditional machine learning and convolutional neural network.

## 1. Introduction

As an advanced technique, myoelectric control is mainly applied in aspects of human-support robots, industrial electronic equipment, or rehabilitation devices, such as surface electromyogram (sEMG)–based wheelchair controller [1,2], exoskeletons [3,4], industrial robots [5], diagnoses, and clinical applications [6,7]. These applications mainly focus on the identification of certain limited pattern types; however, stroke patients have strong demands to improve quality of life by rehabilitation training, which is completed with the assistance of the upper-limb rehabilitation system [8]. Moreover, the upper-limb rehabilitation system that operates with the active participation of the human brain brings great benefits [9,10]. Therefore, it is vital to recognize more types of upper-limb movements because the more upper-limb motion commands can be classified, the more the dexterity of the rehabilitation system that can be gained to reinforce the rehabilitation effect of stroke patients. Thus, this paper intends to investigate the sEMG-based identification of upper-limb locomotion for the rehabilitation system.

As a kind of random bioelectrical signal, sEMG signals are weak, low-frequency, and vulnerable to interference, often accompanied by artifacts, high dimension, and non-stationary problems [11]. Due to the significant differences among different individuals in age, gender, and physique, the characteristics of the sEMG become more prominent [12]. This phenomenon leads to the case that the existing algorithms can discriminate fewer kinds of upper-limb rehabilitation motions, and the classification accuracy is easily altered, which dramatically influences the rehabilitation effect of stroke patients. Accurately identifying more types of upper-limb rehabilitation actions is extremely challenging in this stage [13], which has crucial theoretical significance and application value.

To solve the above problems, time–frequency domain techniques are widely adopted in feature extraction [14,15,16,17,18,19,20,21,22,23,24,25,26,27,28,29]. Englehart et al. utilized the short-time Fourier transform and wavelet transform methods and distinguished six types of upper-limb movements. Shi et al. [15] extracted the time-domain and frequency-domain features for four gestures. Ding et al. [16] gathered the wavelet packet transform coefficient, stationary wavelet transform coefficient, and time–frequency features for the recognition of five kinds of actions. Although the manually designed methods can extract the time–frequency features of sEMG signals, only part of the useful features were considered. However, some beneficial time–frequency features were not taken into account, which resulted in the loss of valid time–frequency features for the sEMG signals and cannot satisfy the discrimination of more rehabilitation actions.

Traditional machine learning algorithms combined with the above methods have been widely applied in intention identification [17,18,19,20,21,22,23], but performance and accuracy are unsatisfactory. Therefore, researchers began to combine various deep learning models for intention recognition [24]. Duan et al. [25] adopted the wavelet neural network combined with the wavelet transform, and the accuracy rate for six gestures was as high as 94.67%. Wei et al. [26] proposed a multistream CNN to recognize three public sEMG datasets, indicating that the neural network approach is superior to the traditional neural network method. Discrete wavelet transform [27,28] can decompose sEMG signals into regular time series with different frequency bands. Thus, Wu et al. [29] collected eight channels of sEMG signals and identified ten gestures using LSTM and CNN. However, the deep learning frameworks commonly applied for intention detection face the following problems: (1) some valuable time–frequency features would be lost during the process of feature extraction; (2) more kinds of upper-limb rehabilitation movements would easily influence the performance of classification algorithms.

To sum up, this paper mainly considers increasing the types of rehabilitation movements, extracting more complete time–frequency features of sEMG and heightening the stability of the identification accuracy for the intention recognition system. The contributions of this paper are as follows:

- Data representation stage: considering the validity and integrity of time–frequency characteristics, a multiscale time–frequency information fusion method is proposed to obtain the dynamic, related information of multichannel sEMG signals.
- Feature extraction stage: in this paper, a multiple feature fusion network is designed for the purpose of considering the relevant features of the current layer and cross-layer of CNN and reducing the loss of the time–frequency features for sEMG signals in the process of convolution operation.
- Classification stage: the deep belief network is introduced as a classification model of the multiple feature fusion network to realize the abstract expression and self-reconstruction for time–frequency features of sEMG signals corresponding to more types of upper-limb movements.

**Figure 1:**
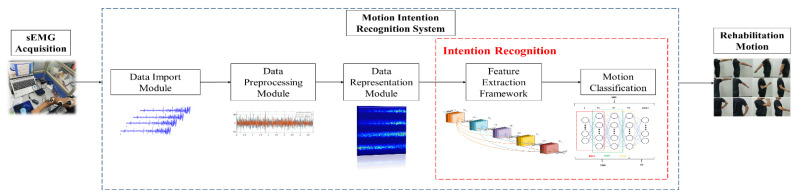
This is the schematic diagram of the proposed intention recognition method in this paper.

Figure 1 shows the overall framework flow chart of this work, and this paper is divided into the following parts. Section 2 describes the rehabilitation actions’ design and sEMG acquisition, meanwhile, the data representation method and the architecture of the proposed MFFN are mainly introduced. Section 3 verifies the performance of the MFFN and then visualizes the learning effect. Finally, the conclusions are discussed in Section 4.

## 2. Methodology

This paper’s main data representation is based on continuous wavelet transform (CWT) [30,31]. In Section 2.1, data acquisition is introduced in detail. In Section 2.2, the approach of multiscale time–frequency information fusion is proposed. In Section 2.3, the basic frameworks and principles of DenseNet and DBN are introduced. In Section 2.4, a multiple feature fusion network based on DenseNet and DBN is presented, and the selection of network parameters is explained.

### 2.1. Data Acquisition

The electromyogram (EMG) signal is a complex biomedical signal produced during the muscle contraction process, which is influenced by anatomical or physiological properties of the muscles and environmental noises [32]. Therefore, the acquisition of EMG signals is a basic task influencing the next classification of upper-limb motion command recognition. In general, EMG signals can be acquired through either invasive or noninvasive electrodes. The EMG signals collected by invasive needles or wire electrodes are suitable for the study of deep muscle structure. However, due to noninvasiveness and convenient operation, sEMG techniques are mainly used to detect sEMG signals in biofeedback, rehabilitation medicine, movement analysis, and other fields. Thus, sEMG is employed as the intermediary carrier to control the upper-limb rehabilitation system.

**Figure 2:**
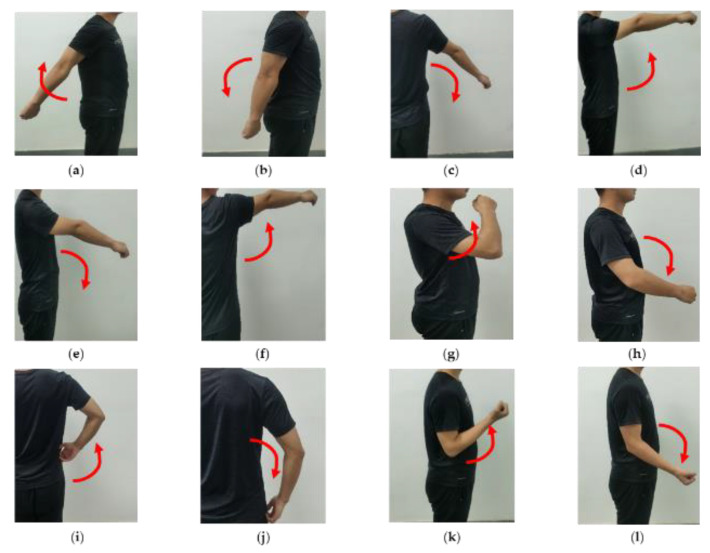
Twelve kinds of upper-limb rehabilitation movements designed for stroke patients are listed as follows: (**a**) posterior extension of shoulder (action1); (**b**) shoulder backstretch return (action2); (**c**) shoulder adduction (action3); (**d**) shoulder anterior flexion (action4); (**e**) shoulder forward flexion return (action5); (**f**) shoulder abduction (action6); (**g**) feeding action (action7); (**h**) feeding return action (action8); (**i**) pant move (action9); (**j**) pant return move (action10); (**k**) elbow flexion (action11); (**l**) elbow extension (action12).

First, considering the principle of human biological structure and the range of motion of each joint, twelve upper-limb rehabilitation exercises, commonly used in rehabilitation training for stroke patients, were designed in this paper, involving single-joint actions (posterior extension of shoulder, shoulder backstretch return, shoulder adduction, shoulder anterior flexion, shoulder forward flexion return, shoulder abduction, elbow flexion, and elbow extension) and compound joint actions (feeding action, feeding return action, pant move, and pant return move); and the action1–action12 are shown in Figure 2.

**Figure 3:**
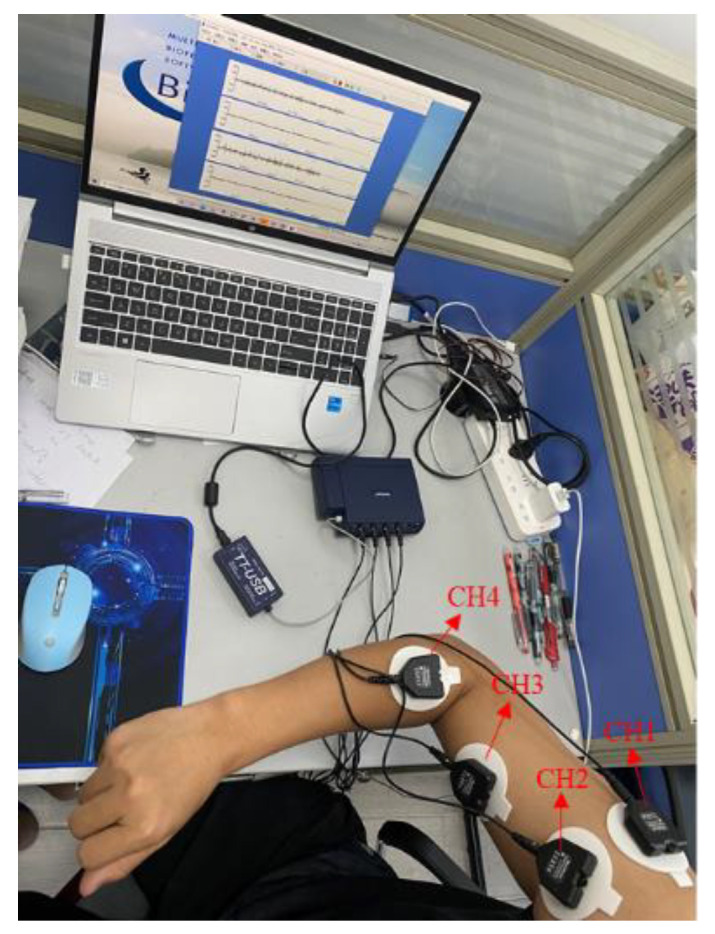
The position of patch electrodes and the sEMG acquisition equipment.

Then, the corresponding muscle groups of the upper limb needed to be determined. Although there are deep-layer and superficial-layer muscles that contribute to the above motions, the sEMG signals are mainly affected by the superficial-layer muscles. Therefore, four superficial-layer muscles were selected to fix the sEMG sensors, CH1 to CH4, which were the deltoid lateral muscle, anterior deltoid muscle, biceps brachii, and radial muscle, respectively, as shown in Figure 3.

To reduce the influence of skin, such as impedance, superficial oil content, and dead cell layer, the surface of the expected muscle groups on the upper limb was wiped with alcohol. Moreover, differential surface electrodes with large distances were utilized to collect sEMG signals after the skin dried. The sEMG signals were recorded with a sampled rate of 2048 Hz using FlexComp Infiniti (Montreal, Canada) acquisition equipment.

Some rehabilitation experts have suggested that one should utilize healthy subjects for initial evaluation objectives, and the sEMG of healthy subjects is an appropriate emulation of the stroke patients’ sEMG. Thereby, in this study, sEMG signals were collected from 14 healthy subjects (male and female) with ages ranging from 20 to 45 years old, with physiques including fat, overweight, normal, thin, and underweight. Each rehabilitation movement lasted for 5 s and was repeated five times. Each action was composed of 47 samples. Therefore, the total dataset consisted of 12 movements × 47 samples × 4 channels × 10,240 = 23,101,360 sampling points.

### 2.2. Data Representation

Before the operation of data representation, the characteristics and attributes of the sEMG signal need to be considered. First, preprocessing of the sEMG signal from its own point of view was executed, and then the data representation operation was performed on the multichannel sEMG signal from the two aspects of the effectiveness and integrity of time–frequency conversion. Therefore, this section consists of two parts: preprocessing and data representation.

#### 2.2.1. Signal Preprocessing

The signal preprocessing is the premise of subsequent data representation. The essence of preprocessing is to weaken the mixed noise in the signal and retain the useful components of the raw signal as much as possible. Thus, it is necessary to carry out a series of preprocessing steps on the raw sEMG signals. In the process of signal acquisition, the sEMG signal is greatly affected by 50 Hz power frequency interference. Therefore, a digital filter was adopted to remove 50 Hz power frequency noise. Moreover, the effective frequency range of sEMG signals is generally distributed in the range of 6~500 Hz, among which researchers have found that most part of the spectrum energy mainly concentrates on 50~150 Hz. Furthermore, the literature [33,34] pointed out that a high-pass filter can remove motion artifact noise while retaining most of the sEMG signal. To sum up, 6 Hz was selected as the cutoff frequency of the high-pass filter in this paper to filter the sEMG signals, which will obtain a more comprehensive sEMG signal without loss of useful information.

#### 2.2.2. Multiscale Time–Frequency Information Fusion Representation

The functional frequency of the sEMG signal itself is 6~500 Hz, so this paper extracts time–frequency features within the corresponding frequency band, and each channel is done by the continuous wavelet transform (CWT), which can greatly reduce the amount of irrelevant data. From the perspective of the time–frequency feature fusion, there is no interaction among multichannel sEMG signals, and the order of input matrices does not affect the classification performance of the network [35]. Therefore, the multiscale time–frequency features could be represented as an RGB image about time, frequency, and amplitude. Finally, the target time–frequency features are extracted as the input of the network. In this paper, the multiscale decomposition module on time–frequency representation (MDTFR) is designed for a single-channel sEMG signal; and the MDTFR module is performed for the multichannel sEMG signals in the valid frequency band. Finally, the time–frequency features of the multichannel sEMG signals are seized comprehensively by fusion operation.

**Figure 4:**
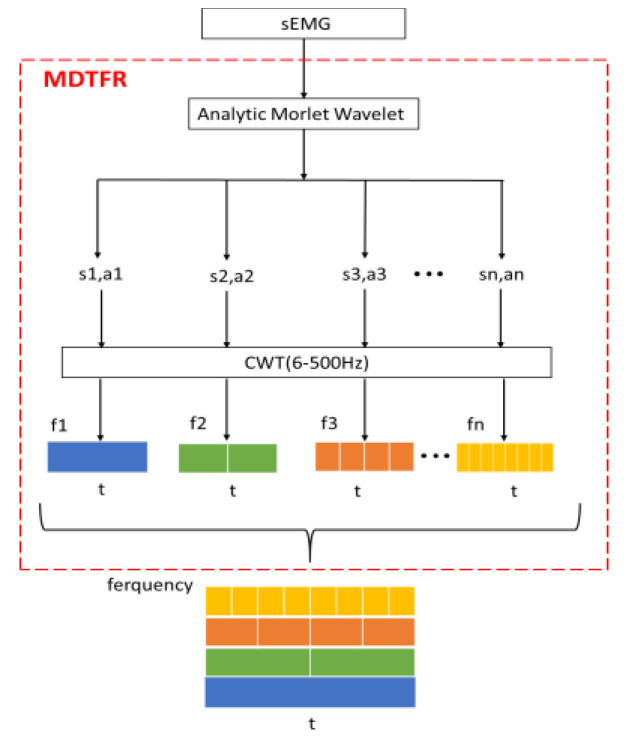
The multiscale decomposition on time-frequency representation for single-channel sEMG.

For the MDTFR module, as shown in Figure 4, the analytic Morlet wavelet, which shows good time–frequency characteristics and has no negative frequency component [36,37], is applied to analyze the time–frequency localization of the received sEMG signals within the effective frequency range. The time–frequency windows that vary with frequency can be provided by adjusting the scale factor, s, and the delay factor, a. Then, the CWT method is used to achieve time subdivision at high frequency and frequency subdivision at low frequency, which can automatically adapt the requirements of sEMG signal analysis so as to focus on the multiscale time–frequency details. This process can be defined as

(1) $$mm[f\left(t\right)]=\{\;S\;|\;S_{i}=CWT_{s_{i},a_{i}}\left(f\left(t\right)\right),\;1\leq i\leq F\text{\}}$$

where $s_{i}$,$\;a_{i}$ represent scale factor and delay factor;  f(t), F correspond to the time signal and its highest effective frequency; $CWT_{s_{i},\;a_{i}}\left(\cdot \right)$ is the continuous wavelet transform under a combination of different $s_{i}\;\mathrm{and}\;a_{i}$. The *CWT* is defined as the inner product of functions f(t) and wavelet $\psi_{s,a}(t)$:

(2) $$CWT_{s,a}\left(f\left(t\right)\right)=\langle f\left(t\right),\psi_{s,a}(t)\rangle =\frac{1}{\sqrt{s}}\int_{-\infty }^{+\infty }f\left(t\right){\bar{\psi }}_{s,a}(t)dt$$

where ${\bar{\psi }}_{s,a}(\frac{t-a}{s})$ is the conjugate function of $\psi_{s,a}(\frac{t-a}{s})$.

**Figure 5:**
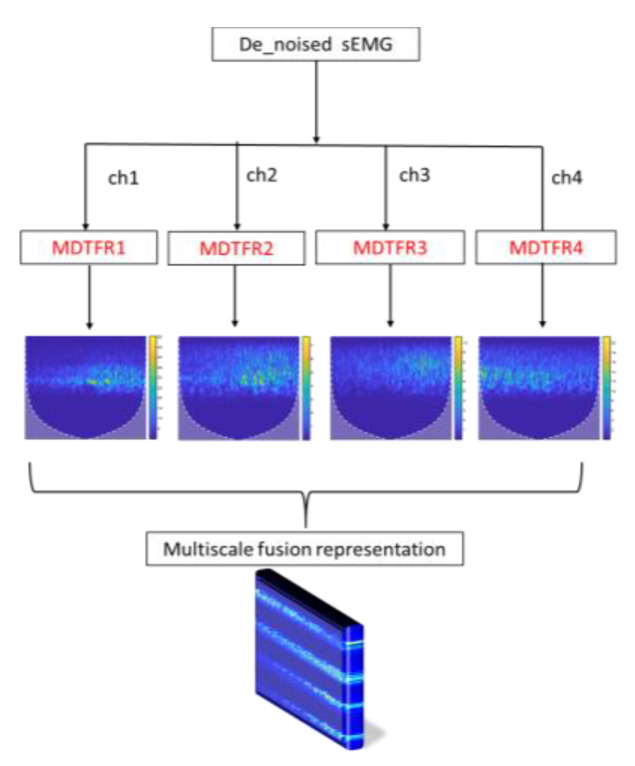
The whole data representation flowchart of multiscale time-frequency information fusion representation (MTFIFR).

The process of MTFIFR is shown in Figure 5. Firstly, the denoised multichannel sEMG signals are processed by the MDTFR module, which aims at collecting the time–frequency features of the surface electromyography data about each channel. Then, these features concerning the valid frequency range of the sEMG signal are fused and represented by concatenating vertically. Finally, the multiscale time–frequency features corresponding to the dynamic information of upper-limb rehabilitation movements are acquired into a three-dimensional RGB image, which can be defined as

(3) $$r=\mathrm{concat}\left({\left[m\left[ch1\left(t\right)\right],m\left[ch2\left(t\right)\right],m\left[ch3\left(t\right)\right],m\left[ch4\left(t\right)\right]\right]}^{T}\right)$$

where *ch*1~*ch*4 correspond to sEMG signals of four channels, and concat(·) represents the longitudinal connection to the feature map.

### 2.3. Components of MFFN

The main data representation method in this paper is based on the above MTFIFR. For the purpose of acquiring complete time-frequency features of sEMG signals for upper-limb movements’ identification, the framework of DenseNet was adjusted to make it more suitable for feature extraction and recognition of sEMG signals in this paper. In the classification stage, in order to improve the adaptability of the network and the stability of identification accuracy for various rehabilitation exercises, this paper realize the bidirectional evaluation of the time-frequency features for sEMG and the corresponding rehabilitation movement by the deep belief network.

#### 2.3.1. DenseNet

**Figure 6:**
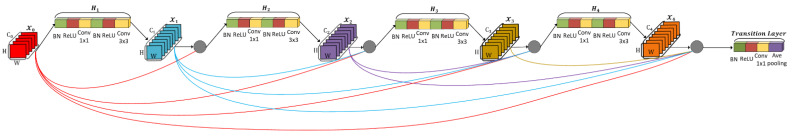
This is the basic framework of dense block and transition layer in DenseNet.

The architecture of a convolutional neural network consists of basic components such as input layer, convolution layer, pooling layer, and classification layer, and most architectures follow this process. In order to ensure maximum information flow among layers of the network, the dense block directly connects the output feature maps of all layers in a feedforward fashion, which reduces the loss of the features in the process of convolution operation [38]. Each layer receives additional input from all previous layers and passes its own feature maps to all subsequent layers, so that the network maintains feedforward characteristics. Figure 6 illustrates this architecture schematically, which is the core component of DenseNet. There is a direct connection between any two layers, and the output of the number L layer is defined as

(4) $$\;x_{L}=H\left(\left[x_{0},x_{1},\;\ldots ,x_{L-1}\right]\right)$$

where *H(·)* represents BN-ReLU-Conv (1 × 1)-BN-ReLU-Conv (3 × 3); $x_{0}$ is the input of the dense block; $\left[x_{1},x_{2},\;\ldots ,x_{L-1}\right]$ refers to the concatenation of the feature maps produced in layers 1,2,…, L−1; and dense blocks are connected by a transition layer, which is generally composed of BN-ReLU-Conv (1 × 1)-Ave pooling (2 × 2). For an L-layer network, the traditional convolutional neural network has L connections, while the DenseNet contains L(L + 1)/2 connections, which significantly raises the transmission and reuse of features. Meanwhile, it is also observed that dense block modules have a regularization effect, which reduces the over-fitting problem of small sample data. Therefore, the DenseNet-based structure is capable of feature extraction for sEMG signals.

#### 2.3.2. The Deep Belief Network

**Figure 7:**
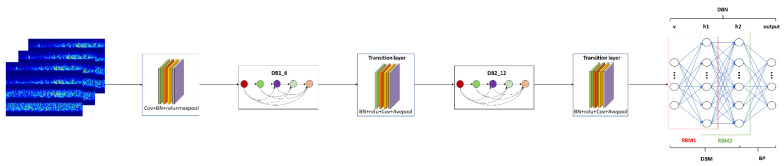
This is the main work framework of this paper, which consists of data representation (MTFIFR) and a learning model (MFFN). The structure of the MFFN, in this paper, is mainly composed of two dense blocks, two transition layers, and a deep belief network.

In the classification stage, the traditional fully connected layer has poor performance due to the complexity and high dimension of sEMG signals caused by individual differences. As a kind of probabilistic generation model, DBN establishes a joint probability distribution between data and labels. The deep belief network (DBN) is composed of a backpropagation (BP) neural network and a depth Boltzmann machine (DBM), and the DBM consists of a series of restricted Boltzmann machines (RBM) stacked in series. Figure 7 illustrates the DBN architecture schematically. The hidden-layer unit of DBN is trained to capture the correlation of high-order data expressed in the visual layer [39], and then features are extracted and reconstructed from the data layer by layer [40,41], and the basic principle of DBN can mainly be defined as

(5) $$P\left(v,h^{1},h^{2},\ldots ,h^{L}\right)=P\left(v{|h}^{1}\right)\cdot P\left(h^{1}{|h}^{2}\right)\cdot \cdot \cdot P\left(h^{L-2}{|h}^{L-1}\right)\cdot P\left(h^{L-1}{|h}^{L}\right)$$

where v is visible layer; $h^{i}$ is the hidden layer of the number i; P(·) is a probability distribution. In this way, the deep belief network extracts and reconstructs data layer by layer and simplifies a complex input pattern, which can promote the network’s ability to represent and generalize data. Meanwhile, the DBN can prevent the network from falling into local optimum to some extent [42]. Thus, the deep belief network is introduced as a classification model to realize the bidirectional evaluation of the time–frequency features of sEMG and the corresponding rehabilitation movement type. Meanwhile, this model can also be applied to capture the intrinsic characteristics of sEMG signals.

### 2.4. Designing Architecture of MFFN

In this paper, the framework of DenseNet is improved to realize the extraction and representation of relevant features for sEMG signals through the convolution operation in the time–frequency domain. First of all, the properties of network input need to be taken into account before designing a feature extraction network. In terms of time–frequency domain features, the denoised sEMG signal, including 10,240 × 4 sample points, is processed by the MTFIFR method to obtain the RGB image, which contains the main time–frequency domain information of multichannel sEMG signals; and the RGB image fed into the convolutional layer is in the form of three-dimensional data. The characteristic of 3D convolution is that the number of channels to the input feature map is equal to the number of convolution kernel channels, and there is no need to slide in the channel direction. Therefore, this paper chose a 3D filter whose kernel size is the number of input channels to fuse the information of each channel, instead of a small size convolution of channels. Secondly, convoluting across time–frequency domain information about sEMG signals will lead to deep learning of the dynamic properties and frequency components hidden in sEMG signals. In this paper, time–frequency domain convolution is realized by the optimized DenseNet. Third, in the classification stage, in order to heighten the recognition algorithm’s performance for various rehabilitation motions, the time–frequency information of sEMG signals is reconstructed through neurons to capture the essential attributes of sEMG and establish a probabilistic approximation model between sEMG and the corresponding rehabilitation movement by the deep belief network.

As shown in Figure 7, the inputs to the network are first convolved through the channels and then placed into the modified DenseNet. Within each dense block, there are a certain number of dense block layers, which are responsible for fusing the features of sEMG signals at each learning stage and splicing together the learned time–frequency features of sEMG signals. Meanwhile, in order to prevent too large or too small a range of features from affecting the convergence speed of the network, the normalization layers and activation layers are added after the network input layer [43,44,45]. Subsequently, the time–frequency features of the sEMG signals are concatenated and sent to the transition layer, which controls the feature map’s size and considers the compatibility of the feature map with the computation burden of the network [46]. For the purpose of acquiring more comprehensive features about rehabilitation actions [47], the above operations are performed repeatedly to gain the deeper features of sEMG signals. After repeating this phase, the feature map is sent to the deep belief network for the identification of upper-limb movements. Finally, various parts in the MFFN such as the convolution layer, dense block module, transition layer, and deep belief network structure are selected by the cross-validation method, which will be demonstrated and discussed in the experimental results.

## 3. Experimental Results and Discussion

For experiments, the signal preprocessing and network construction that were carried out in MATLAB 2020b environment used 16 GB RAM and CPU of Intel^®^ Core^TM^ i7-10750H CPU @2.60 GHz 2.59 GHz. As for deep learning, the NVIDIA GeForce GTX 1660 Ti GPU with 7.9 GB of RAM was employed. The whole process is supported by the Windows 10 operating system.

### 3.1. Parameter Selection

Each sample has a total of 10,240 × 4 sample points. Therefore, the appropriate input size was firstly selected for the network framework. Then, the key parameters of MFFN such as the convolution kernel size, the number of dense blocks, and DBN structure were optimized by cross-validation. Finally, the training parameters such as learning rate and iteration number were adjusted by the grid searching method, and the cross-entropy and Adam were chosen as the loss function and optimization algorithm of the MFFN. Based on the above analysis, parameter selection and tuning of the MFFN were executed, and detailed information is shown in Table 1.

### 3.2. Experimental Comparison and Analysis

In terms of dataset splitting, this paper took the data of 12 rehabilitation movements for each subject as a whole, and then randomly selected all rehabilitation movement data for some of the subjects among all subjects as the test set, and the rest as the training set. Several experiments were carried out to evaluate the performance of the network framework. The experiment was mainly executed from two dimensions. In the vertical aspect, the performance of DBN and traditional fully connected layer as MFFN classification model was compared. In the horizontal aspect, the recognition performance of different network frameworks for 12 rehabilitation movements were contrasted, and the specific schemes and results are given subsequently. Finally, this paper analyzed and discussed the performance of the proposed method.

#### 3.2.1. Longitudinal Contrast Experiment

In order to verify the ability of the DBN to capture time–frequency features of sEMG signals, the performance of traditional fully connected layer and DBN structure as a classification model of the MFFN were compared respectively; and the architectures were selected by the cross-validation method.

In Table 2, the experimental results show that the proposed method can effectively improve the accuracy of the intention recognition system in each test, which proves that the DBN has a better effect on the abstract representation and reconstruction of time–frequency features for sEMG signals. Furthermore, the accuracy of the DBN model with the MFFN is 1.64% higher than that of the fully connected layer in an average sense. Thus, the results indicate that the proposed DBN as the classification layer of MFFN is more effective in upper-limb movement identification.

#### 3.2.2. Transverse Contrast Experiment

To validate the performance of the proposed time–frequency representation method and the MFFN framework in motion intention classification, the proposed method was compared with traditional machine learning algorithms and common deep learning network frameworks. The identification results of different frameworks and the proposed MFFN for 12 rehabilitation movements are shown in Table 3, and the average accuracy is better than in the previous approaches. The results of the first two columns are obtained by using traditional, manual-design time–frequency features and machine learning approaches; and the MFFN increases by 7.35% and 3.63% versus LDA (linear discriminant analysis) and SVM (support vector machine). It is obvious that the identification effect of the MFFN is more noticeable. Moreover, the first three methods in the table all use the time–frequency features of sEMG, while we choose the MTFIFR approach as the data representation. Therefore, compared with the previous three ways, the presented method retains more complete time–frequency features of sEMG signals, does not lose too much intermediate information in the convolution operation, and learns more relevant features. At the same time, the classification accuracy of the test for number 2, 5, 8 in the table are slightly less than in the LCNN (long short-term memory network and convolutional neural network) [26] method, but, overall, the accuracy of the other items was boosted, which illustrates the advantages of continuous wavelet features combined with MFFN in motion intention recognition. Compared with the end-to-end network frameworks including G_CNN (convolutional neural network with gate) and LCNN, the identification effect of the MFFN is superior, indicating that the proposed time–frequency representation method combined with MFFN is more effective in rehabilitation movement classification.

#### 3.2.3. Analysis and Discussion

**Figure 8:**
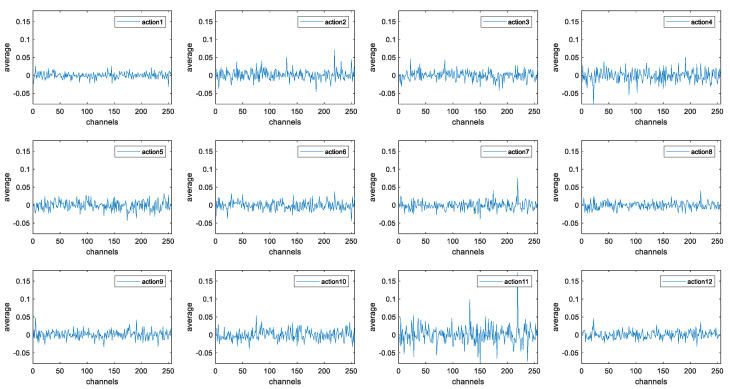
The extraction effect of multiple feature fusion network for the time–frequency features of sEMG signals corresponding to 12 kinds of upper-limb rehabilitation movements.

For analyzing the performance of the MFFN, we first extracted the features of Transition Layer (2) for the proposed feature extraction framework and carried out an average operation on each feature map channel. The visualization results are shown in Figure 8, and the data distribution of each action from action1 to action12 has no significant mutation and remains within a certain range, which proves that the MFFN can extract stable time–frequency features of sEMG signals. At the same time, the data of action1, action2, and other actions have great differences in the amplitude change process, and the corresponding rehabilitation movements can be well distinguished through the dynamic change process of the data, illustrating that MFFN can better extract the useful time–frequency features of complex sEMG signals.

**Figure 9:**
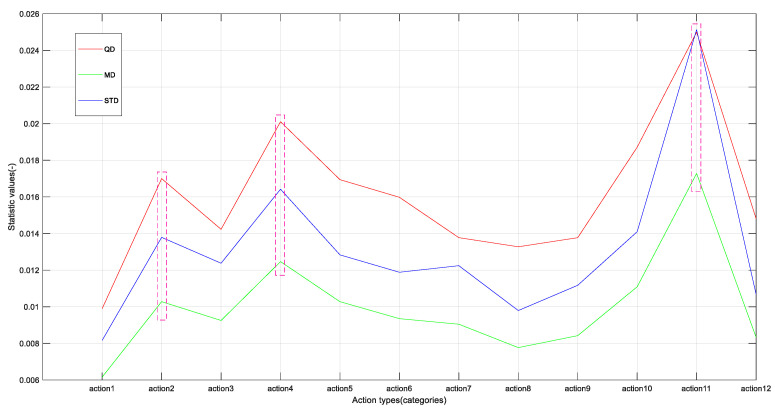
Evaluating the mutation of the extracted feature for each class action by statistics (QD, MD, and STD).

To evaluate the mutation of the extracted feature for each class of action and to measure the distinctions in Figure 8, first, the statistics of quartile difference (QD), mean difference (MD), and variance (STD) were introduced to evaluate the mutation of the extracted feature for each class of action on the basis of Figure 8, in which,

(6) $$QD\;=Q_{3}-Q_{1}$$

(7) $$MD=\frac{\sum_{i=1}^{n}\left|x_{i}-\bar{x}\right|}{n}$$

(8) $$STD=\sqrt{\frac{\sum_{i=1}^{n}{\left(x_{i}-\bar{x}\right)}^{2}}{n-1}}$$

where $Q_{1}$ is the next quartile; $Q_{3}$ denotes the upper quartile; $x_{i}$, $\bar{x}$, and n represent the *i*-th element, average, and length of the data, respectively. The greater the three above, the greater the fluctuation in the data and vice versa. The results are shown in Figure 9 as follows. On the whole, the trend of three statistics describing the mutation of 12 kinds of action features is consistent. At the same time, it can be seen that the corresponding statistical values of action2, action4, and action11 are larger, and those of some actions are smaller, e.g., action1 and action8. This shows that there are great differences in the process of feature mutation corresponding to the 12 types of actions, which indicates that the features extracted by the proposed method are beneficial to the classification of multiple actions from the aspect of mutation degree.

**Figure 10:**
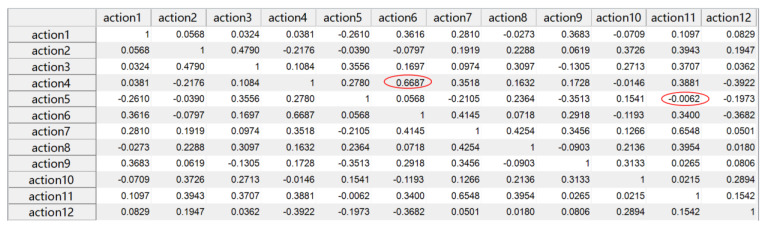
The correlation coefficient matrix among the 12 types of actions.

Then, the correlation coefficient method was used to analyze the correlation of the features extracted from the 12 types of actions to observe the distinctions among actions. The expression of correlation coefficient is

(9) $$\rho_{X,Y}=\frac{con\left(X,Y\right)}{\sigma_{X}\sigma_{Y}}=\frac{E\left[\left(X-EX\right)\left(Y-EY\right)\right]}{\sigma_{X}\sigma_{Y}}=\frac{E\left(XY\right)-E\left(X\right)E\left(Y\right)}{\sqrt{E\left(X^{2}\right)-E^{2}\left(X\right)}\sqrt{E\left(Y^{2}\right)-E^{2}\left(Y\right)}}$$

where con(·) is the covariance, σ is the standard deviation, and E denotes the mathematical expectation; the correlation coefficient takes values in the range [−1,1], and in general, the closer the correlation coefficient is to 1, the more positive the correlation is; the closer it is to −1, the more negative the correlation is; and the closer it is to 0, the less relevant it is. The results are shown in Figure 10. It is obvious that the correlation coefficients in red marked the maximum (the correlation coefficient between action4 and action6) and the closest to zero (the correlation coefficient between action5 and action11) are 0.6687 and −0.0062, respectively, indicating that there is a certain correlation between action4 and action6, and there is a very weak correlation or no correlation between action5 and action11.

**Figure 11:**
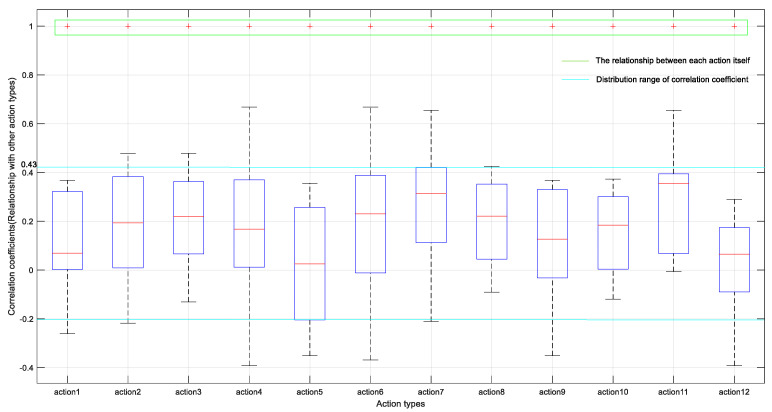
Box plot of correlation coefficient distribution among 12 action types.

To further compare the distinctions in features across action1–action12, the distribution of the correlation coefficients was visualized by means of a box plot, which is a method of describing the distribution of data using five statistical values (minimum, first quartile, median, third quartile, maximum, accompanied by outlier points). The horizontal axis indicates the action types, and the vertical axis represents the distribution of the correlation coefficients, where the green part denotes the correlation coefficient between each action class and itself, with a constant value equal to 1; each box means the correlation among the current action class and the rest. The corresponding results are shown in Figure 11, it can be seen clearly that the overall correlation coefficient is mainly distributed between −0.2 and 0.43. At the same time, there are significant differences in the position of each box, which shows that there is a relatively weak correlation among 12 kinds of actions, that is, there are big distinctions among the features of different types of actions, which proves the effectiveness of the proposed method in this paper.

**Figure 12:**
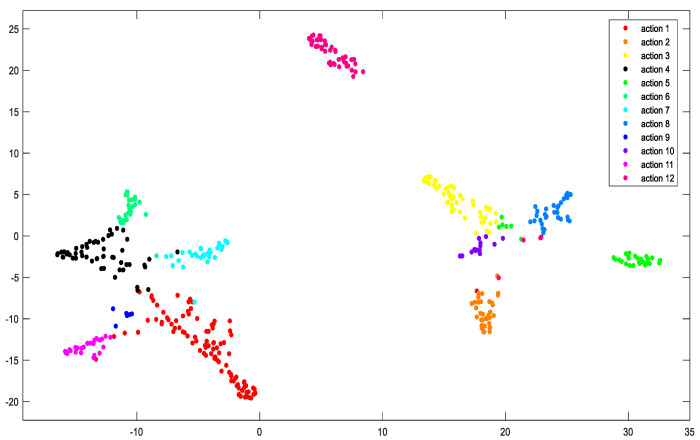
Feature distribution of 12 types of rehabilitation motions, obtained by visualizing the last layer features of DBN using the t-SNE method.

Then, this paper further analyzed the learning effect of DBN as the classification model of the MFFN. As shown in Figure 12, the output features of the DBN model are reduced in dimension and visualization by the t-SNE (t-Distributed Stochastic Neighbor Embedding, which returns a matrix of two-dimensional embeddings of the high-dimensional rows of the input) approach. It can be observed in the distribution of features that although there is a small amount of data overlap, the 12 classes can be well distinguished. For example, there is a large distance among action1, action2, and action6, which indicates that DBN introduced as the classification model of the MFFN can effectively capture the time–frequency features of sEMG signals and perform well in recognition of more types of rehabilitation actions.

**Figure 13:**
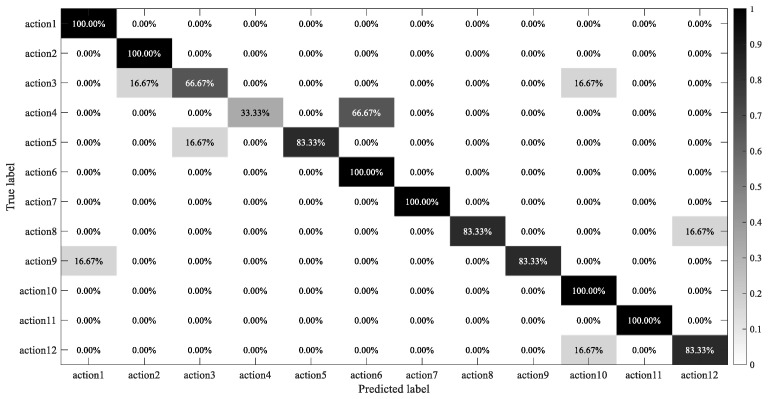
Confusion matrix of motion classification.

Finally, the identification results of 12 rehabilitation exercises were presented in the form of a confusion matrix, as shown in Figure 13. It is relatively serious that action4 was mistaken as action6, and the main reason may derive from the high similarity of movements that are mainly driven by the same muscles. As a result, the time–frequency features of sEMG signals are not obvious enough to distinguish these two actions, which also verifies the overlap of a small number of features in Figure 12. However, most actions, such as action1, action2, action6, action7, action10, and action11, can be classified well, which demonstrates that the proposed time–frequency representation method combined with MFFN is more effective in recognizing more types of rehabilitation movements.

## 4. Conclusions and Future Outlook

In this research, first, the data representation method can fuse more complete time–frequency dynamic information about sEMG signals. At the same time, most of the invalid frequency domain information of the sEMG signal can be effectively suppressed, and the valid frequency domain information can be well preserved, which is beneficial to boost the learning efficiency. Then, this paper mainly proposes the application of a multiple feature fusion network in sEMG signals. The MFFN does not learn the time–frequency features of sEMG signals from the depth and breadth of the architecture but strengthens the network through reusing or fusing features. The feature loss in each part of the network and the number of parameters are reduced. Finally, the deep belief network is introduced as a classification model of the MFFN in the classification stage, which can realize the bidirectional evaluation of the time–frequency features of sEMG and the corresponding rehabilitation movement. Meanwhile, the deep belief network can capture the intrinsic properties of surface EMG signals, which is conducive to improving the generalization and accuracy of the multiclassification system. The final experimental results and visualization analysis prove that the multiple feature fusion network can effectively distinguish more types of upper-limb rehabilitation movements. Compared to SVM and other CNN frameworks, the multiple feature fusion network can produce better results, demonstrating that the multiple feature fusion network structures make sense for sEMG signals.

However, in the current architecture of the network there mainly exists a problem that needs to be verified and solved, that is, there may be redundant features in the process of time–frequency feature extraction from sEMG signals. To sum up, the MFFN provides an effective network framework for extracting more stable and valuable features from sEMG signals. In the future, we will integrate the above issue further to enhance the performance of the feature fusion network framework and apply it to the field of rehabilitation medicine.

## Figures and Tables

**Table 1:** This is the network configuration of the proposed architecture (MFFN).

Layers	Output Size	Multiple Feature Fusion Network
Input Layer	224 × 224 × 3	--
Convolution	112 × 112 × 64	filtersize: [7,7], numfilters: 64, stride 2, padding: [3,3,3,3]
BN	112 × 112 × 64	channels: 64
ReLu	112 × 112 × 64	--
Pooling	56 × 56 × 64	[3,3] max pool, stride: [2,2]
Dense Block (1)	56 × 56 × 256	[BN, ReLu, 1 × 1 conv, BN, ReLu, 3 × 3 conv] × 6
Transition Layer (1)	56 × 56 × 256	BN
	56 × 56 × 256	ReLu
	56 × 56 × 128	[1,1] conv
	28 × 28 × 128	[2,2] average pool, stride 2
Dense Block (2)	28 × 28 × 512	[BN, ReLu, 1 × 1 conv, BN, ReLu, 3 × 3 conv] × 12
Transition Layer (2)	28 × 28 × 512	BN
	28 × 28 × 512	ReLu
	28 × 28 × 256	[1,1] conv
	14 × 14 × 256	[2,2] average pool, stride 2
Classification Layer	1024 × 1	1024 × 50,176 RBM
	1500 × 1	1500 × 1024 RBM
	12 × 1	12D Fully Connected, softmax

**Table 2:** Comparison results of DBN and FC (fully connected) as a classification module of the MFFN.

Methods	MFFN_FC	MFFN_DBN
subject 1, subject 2	83.94%	86.10%
subject 3, subject 4	63.23%	65.11%
subject 5, subject 9	79.17%	79.37%
subject 6, subject 10	75.00%	77.78%
subject 3, subject 9	62.33%	65.89%
subject 1, subject 4	76.39%	77.39%
subject 7, subject 10	62.69%	62.78%
subject 2, subject 5	83.33%	85.10%
subject 6, subject 7	64.17%	66.56%
subject 7, subject 9	68.28%	68.83%
AVE	71.85%	**73.49%**

MFFN_FC: multiple feature fusion network proposed in this paper combined with fully connected layer. MFFN_DBN: multiple feature fusion network proposed in this paper combined with deep belief network.

**Table 3:** Comparison results of different frameworks for recognizing 12 rehabilitation movements.

Methods	LDA [14]	SVM [48]	G_CNN [49]	LCNN [26]	Proposed Method
subject 1, subject 2	80.26%	85.03%	85.29%	80.11%	**86.10%**
subject 3, subject 4	52.43%	60.29%	62.59%	**65.29%**	65.11%
subject 5, subject 9	70.21%	72.98%	78.39%	75.22%	**79.37%**
subject 6, subject 10	65.16%	69.26%	75.00%	70.92%	**77.78%**
subject 3, subject 9	59.98%	65.53%	65.39%	**65.98%**	65.89%
subject 1, subject 4	70.56%	72.20%	75.65%	74.61%	**77.39%**
subject 7, subject 10	60.92%	60.89%	61.85%	61.79%	**62.78%**
subject 2, subject 5	78.23%	78.29%	80.59%	**85.49%**	85.10%
subject 6, subject 7	62.51%	64.93%	65.29%	63.46%	**66.56%**
subject 7, subject 9	61.09%	**69.22%**	67.92%	62.34%	68.83%
AVE	66.14%	69.86%	71.80%	70.52%	**73.49%**

## Data Availability

Not applicable.
